# The FIFA World Cup Qatar 2022 Sustainability Strategy: Human Rights Governance in the Tripartite Network

**DOI:** 10.3389/fspor.2022.809984

**Published:** 2022-05-23

**Authors:** Andrew O'Rourke, Eleni Theodoraki

**Affiliations:** School of Public Health, Physiotherapy and Sports Science in University College Dublin, Dublin, Ireland

**Keywords:** policy networks, human rights, sustainable development, network governance, FIFA 2022 World Cup Qatar

## Abstract

The Fédération Internationale de Football Association (FIFA) has faced constant accusations of human rights violations associated with World Cup Qatar 2022, with prominent media coverage and international football team demonstrations. This study aims to analyze and discuss the approach taken by the tripartite policy network of actors, namely FIFA, Qatar's Supreme Council (SC), and the Local Organizing Committee (Q22) for the creation of the the FIFA World Cup Qatar 2022 Sustainability Strategy (hereafter WCSS22) published in January 2020. The WCSS22 represents the first time FIFA has clearly articulated its responsibility in connection with impacts that are linked to the construction and operation of World Cup stadia and facilities, in line with the United Nations Guiding Principles on Business and Human Rights (UNGPs). The strategy was also the first to be planned and delivered jointly by FIFA, the SC, and the Q22. Qualitative Content Analysis (QCA) of documents associated with the WCSS22 was performed to answer the following research questions: a) What are the recurrent features of the policy formulation and design process and what role do the UNGPs play? b) How do FIFA and the other policy actors contribute and position themselves in relation to human rights? c) What form did governance (interdependence, interactions, regulated rules, and steering) take in the policymaking process? The study establishes that there are four recurrent features of policy formulation and design: 1) a collective, systematic, and diverse policymaking approach, 2) emphasis on leveraging internal resources and external input, 3) the building foundation of best practice principles, guidelines, strategies, and existing initiatives, and 4) inconsistency on decision-making and accountability measures. FIFA contributes to policymaking primarily through their existing statutes, human rights policy, and commitments to mitigate negative human rights impacts. Furthermore, specified actions, and mechanisms for construction workers' living and working conditions and recruitment processes are articulated by the SC, who take a more prominent role in worker initiatives. Q22, although involved in collective action, and participating in workers welfare, takes a more peripheral role in the policymaking process. In conclusion, it was found that the tripartite policy network of actors represents a participant-based governance approach with cohesive policy formulation, varied resources at their disposal, inconsistencies in accountability measures and with the lead network role dependent on specific actor initiatives and commitments.

## Introduction

Circa $200 billion has been invested for FIFA World Cup Qatar 2022 with between 500,000 and 1.5 million foreign workers employed (Ganji, 2016). However, repeated accusations of human rights violations of migrant construction workers for the tournament infrastructure, their exploitation, and deaths of migrant construction workers for the tournament infrastructure, have dominated the associated literature (Renkiewicz, 2016; Amis, 2017; Heerdt, 2018; Heerdt and Duval, 2020). Prominent media coverage has subsequently resulted as well as international football team demonstrations (BBC, 2021; The Guardian, 2021). Despite being a non-state actor, the newly established regulatory and policy framework of Organization for Economic Cooperation and Development (OECD) Guidelines for Multinational Enterprises and the UNGPs identify FIFA's role as bearer of human rights responsibilities (Kirschner, 2019). The WCSS22, the first to be formulated jointly by FIFA, the host country SC, and Q22 (FIFA, 2020a), applies to all functional areas and projects involved in the preparations for and staging of the tournament, along with post-event activities (FIFA, 2020b). The strategy details policy commitments and objectives under five pillars: human, social, economic, environment and governance, with articulation of responsibility for the first time by FIFA in connection with human rights, in line with the UNGPs (FIFA, 2020e). Also highlighted in the WCSS22 is reference to the specific SDGs and targets. This includes Goal 8: “*Promote sustained, inclusive and sustainable economic growth, full and productive employment and decent work for all.”* Referring to this goal, the WCSS22 states the intention to ensure decent working and living conditions and fair recruitment for workers engaged in the construction of, and provision of services for, FIFA World Cup 2022™ sites.

Developed by Professor John Ruggie, the UNGPs are considered the global standard of expected conduct for all companies regarding human rights policy, providing clarity and predictability and responsibility to respect human rights (Davis, 2012; Thuer, 2017). The principles articulate states' obligations to human rights and freedoms, the role of business enterprises to respect rights in line with the drive toward corporate social responsibility; and the need for remedies when rights and obligations are breached (United Nations, 2011).

In the aftermath of FIFA's commissioned report from John Ruggie and Shift Ltd. to analyze its internal governance in 2016 (Ruggie, 2016), a FIFA Governance Committee and FIFA Human Rights Policy was established to address human rights, as part of a sustainability program which has undertaken various activities to promote awareness and address negative impacts (FIFA, 2017). Several commentators suggest FIFA's recent prioritization of human rights has quickly become central to its newly stated institutional mission and identity (Buhmann et al., 2019; Krech, 2020). Others claim FIFA have developed policies aligning with the UNGP principles to protect themselves from accusations of compliance, shying away from accepting responsibility for monitoring, and acting upon, human rights abuses (Næss, 2018; McGillivray et al., 2019). The UNGPs provide clarity in outlining the human rights duties of governments and private actors are independent of each other (OHCHR, 2011); however, not all governments may be willing or able to enforce international human rights standards effectively (Mares, 2011; Wettstein, 2015). To understand Qatar and its relationship with the FIFA World Cup 2022 event, Hayajneh et al. (2017) and Millward (2017) claim that the recent social and political context of the country must be understood first. A policy commitment, as statement of responsibilities or expectations with regard to respect for human rights across activities and business relationships (Shift and Mazars, 2015), has evidently been made to sustainable development as part of the state's ongoing development within the framework of the Qatar National Vision (QNV) 2030 strategy (GCO, 2008). The state of Qatar, however, continues to face the challenge of social inclusion and human and labor rights equality with persistent accusations of abusive labor practices (Talavera et al., 2019).

## Theoretical Framework

Public policymaking and delivery are being increasingly shaped by new and diverse organizational and structural configurations such as networks and strategic collaborations with varied modes of governance (Keast et al., 2006). Furthermore, policy is rarely based on shared meanings between different actors that are active in the changing nature of network governance (Bevir and Richards, 2009a). Unexplored to date from a research perspective, and potentially a key determinant in the analysis of the WCSS22, is the interaction, interdependence, and negotiation of a complex set of actors, and relevant impact in the policy process for the WCSS22 (Wolde, 2019). As the WCSS22 was the first FIFA World Cup Sustainability Strategy of its kind to be constructed jointly through multiple actors, with articulation of UNGP alignment for the first time, the question emerged - how was policy collectively formulated and designed to address human rights and what role did FIFA play in the process? Given the tripartite configuration of actors, a policy network perspective is a valuable tool to analyze the relations among actors in a policy area and further explain the reasons and results of the existence of relationships (Luo et al., 2011). Policy networks consist of governmental and societal actors whose interactions with one another give rise to policy (Bevir and Richards, 2009b). They are viewed as a purposive course of action followed by an actor or set of actors in dealing with a problem or matter of concern (Miller and McTavish, 2013). Policy networks, it is argued, can have two tiers, a core, and a periphery, with a clear distinction between members with and without access to resources and influences (Rhodes and Marsh, 1992). Policy network analysis attempts to explain policy development by examining networks of actors concerned with a given policy problem, across the public and private sectors and throughout different levels of governance (Mikkelsen, 2006). This study employs an interorganizational perspective, which helps reveal private and public configurations that exist in policy formation, identifying the position on human rights of FIFA and other actors and the ways in which networks influence actors' capacity to use their resources, creating policymaking mechanisms (Thatcher, 1998). Application of this perspective also allows for the identification of relations or patterns of strategic actions between a set of actors in policy formulation (Kenis and Schneider, 1991).

Understanding the complex interactions of policy measures can play a significant role in policy design and analysis (Taeihagh, 2017). Policy design can be defined as an activity to pursue policy goals through gathering and applying knowledge, conducted in specific spaces, dependent on contextual conditions, and can be constrained by the previous design (Capano, 2018). Designing successful policies requires both substantive instruments, a set of alternative arrangements capable of resolving or addressing a policy problem, as well as a procedural instrument, and a set of activities related to securing some level of agreement among those charged with formulating, i.e., network structure (Howlett and Mukherjee, 2014; Mukherjee et al., 2021). The development of systematic approaches and tools is also required for the exploration of design spaces and diversity of preferences of different stakeholders (Taeihagh, 2017). Objectives, however, must be concretized in a set of specific targets or measures which allow policy resources (Olsson and Hysing, 2012) to be directed toward goal attainment (Howlett, 2009). The research objective concerning policy creation for this study is to identify the recurrent features of the policy formulation and design process and the role that the UNGPs play in relation to Qatar 2022.

An actor can be viewed as an organization that can make decisions and acts in a coordinated way (Hermans and Cunningham, 2013). Within a policy network, each actor has an interest and capacity to help determine its character (Luo et al., 2011), with strategies taken to use the network to satisfy their needs, interests, and goals, and manage their interdependencies (Van Waarden, 1992). Preferences and positions taken by actors can also translate values into a preference ordering over specific solutions (Hermans and Cunningham, 2013). The concept of design coalitions indicates relational structures of actors who advocate for specific policy design elements during the design process (Haelg et al., 2020) and adopt collective decisions to jointly deploy their resources (Füg, 2011). Finally, the term “policy community” concerns those actors who exchange resources to balance and optimize their mutual relationships, while “issue networks” represent a range of interests, fluctuating interaction, and access for the various members (Rhodes and Marsh, 1992). The study's related objective is to find how FIFA and the other actors contribute and position themselves in relation to human rights in the WCSS22 strategy documents.

Governance is concerned with a complex matrix of interactions and interrelations between different actors, and between different sets of ideas and practices, and thus has significant implications for policymaking (Zafarullah, 2015). Within the network context, governance can arguably be interpreted through the conceptual model of interdependence between organizations, interactions caused by the need to exchange resources, game-like interactions regulated by rules negotiated and agreed by network participants, as well as network steering activities to manage and attain greater central control (Rhodes, 2007; Fawcett and Daugbjerg, 2012; Klijn and Koppenjan, 2012). In network analytical approaches, a common objective is to describe relational configurations (Provan and Kenis, 2007), with network governance a form of organizational alliance in which relevant policy actors are linked together and likely to identify and share common interests (Kim, 2006). Network management strategies also include organizing joint research and fact finding (Klijn and Koppenjan, 2012). Participant governed networks can be highly decentralized, involving equal network members interaction. While lead organization-governed networks occur when one organization has sufficient resources and legitimacy to play a lead role (Provan and Kenis, 2007), with elements of “networked power” in setting the agenda for decision-making (Castells, 2011, cited in Millward, 2017, p. 761). In addition to assessing the contribution and position taken by the actors in relation to human rights, this study also sought to ascertain what form of network governance took place in this particular policymaking process (Rhodes, 2007; Fawcett and Daugbjerg, 2012; Klijn and Koppenjan, 2012) using the conceptual model (interdependence, interactions, regulated rules, and steering) identified within the literature.

## Methodology

As the aim of this research was to understand and analyze the tripartite policy network of actors involved in a specific policymaking process, a qualitative approach was adopted. An interpretivist ontological position was taken to identify and understand the nature of the collaboration, using tripartite policy network processes and interactions (Bevir and Richards, 2009a). The strength and power of the interpretivist approach lies in its ability to address the complexity and meaning of situations (Black, 2006). Qualitative Content Analysis using interpretivist methods also assumes the meaning of text data is subjective and requires external information about the originator of the text (Lacity and Janson, 1994). Ontology, as a concept, is concerned with understanding the existence of, and relationship between, different aspects of society such as social actors and structures, with social ontology related to ascertaining the nature of social entities (Al-Saadi, 2014). Therefore, the qualitative approach and position taken allowed for meanings and understandings to be determined.

To provide a richer understanding of networks of actors involves methodologies, such as textual analysis, as a way of recovering meanings (Bevir and Richards, 2009b). Documents were used as the unitary source of data for this study given their usefulness as a standalone method for specialized forms of qualitative research producing rich descriptions of a single phenomenon or event (Bowen, 2009). Documents, including government plans and reports, can also provide useful information for researchers to address a variety of questions, with policy another important domain to generate network data (Hu, 2015). The examination of documents for this study allowed the researcher to understand the interorganizational collaboration through strategic alliance, management practices and policy making (Hu, 2015). Data was collected using the strategy documents associated with the publication of the WCSS22. The main documents used for analysis were the “FIFA World Cup Qatar 2022 Sustainability Strategy” (WCSS22) (FIFA, 2020b) and “The Development of the FIFA World Cup Qatar 2022 Sustainability Strategy” [hereafter DWCSS22] (FIFA, 2020a), which was developed as a supporting document, providing an overview of the strategy development process (FIFA, 2020a). Other documents used to provide context were the “For the Game, For the World” report (Ruggie, 2016) “Executive Summary for FIFA World Cup Qatar 2022 Sustainability Strategy (FIFA, 2020f),” “FIFA World Cup Qatar 2022 First Sustainability Progress Report (FIFA, 2020c),” “UNGPs” (OHCHR, 2011), and “FIFA World Cup Qatar 2022 Sustainable Sourcing Code (FIFA, 2020d).”

Based on the areas identified for analysis in the literature review, a text extraction plan was created which would form the basis of the study. Data was recognized within the strategy documents that would be of most relevance to the topic, to develop findings and create a structure for analysis. This included all sections, including annexes, of the DWCSS22 and forewords, introduction, strategy overview, pillars (human, social and governance pillars), alignment with the UN Sustainable Development Goals, and annexes for the WCSS22. The economic and environment pillars for the WCSS22, while considered as reference guides, were not extracted for analysis in the study due to a lack of relevance to the topic of human rights.

Qualitative approaches allow for exploring new or understudied network phenomena (Hollstein, 2014) with Qualitative Content Analysis (QCA) used as “a research method for the subjective interpretation of the content of text data through the systematic classification process of coding and identifying themes or patterns” (Hsieh and Shannon, 2005, p. 1278). QCA also involves the process of distilling words into fewer content-related categories (Elo and Kyngäs, 2008). Inductive (conventional) QCA is used when there is lack of, or limited, previous theories or research findings, while deductive (directed) QCA is used when some views, previous research findings, theories, or conceptual models regarding the phenomenon of interest exist (Armat et al., 2018). Using a directed approach, the categories created can be used to refine, test, or further develop conceptually the theoretical framework (Hsieh and Shannon, 2005; Assarroudi et al., 2018).

QCA was adopted for this study using inductive and deductive methods, both concurrently and independently (Armat et al., 2018). The text from the DWCSS22 and WCSS22 were firstly added line by line to excel spreadsheets. An initial inductive approach was taken with open coding of the text which was then broken down further until the text fell into themes and then subcategories. These subcategories were then analyzed to establish four main categories (Hsieh and Shannon, 2005; Elo and Kyngäs, 2008) and summarized to create the recurrent features of the policy formulation and design process and role of the UNGPs. Through an iterative process (Bowen, 2009) and using a mixed methods approach, key segments of actor contribution were separated, using color coding, and grouping (inductive), extracted onto additional spreadsheets, and analyzed using the subcategories created (deductive) (Hsieh and Shannon, 2005; Elo et al., 2014; Assarroudi et al., 2018). Key human rights positioning pillars (see Appendix A) were established for each actor using the content summarized for the findings. To ascertain the governance form, a deductive approach was taken, using the key human rights positioning pillars for each actor, and aligning them with definitions of the conceptual model components (interdependence, interactions, rules and regulations and steering). These components were applied as the structured categorization matrix to form a summary of the key findings (Elo and Kyngäs, 2008; Elo et al., 2014; McGillivray et al., 2019).

The text data was then reviewed iteratively to ensure no relevant categories or topics were missed as part of the process while hand notes were also used consistently throughout the coding process. Both manifest and latent content approaches were applied to ascertain a deep understanding of the data (Assarroudi et al., 2018). The approach of de-contextualization, re-contextualization, categorization, and compilation was also adopted to ensure validity and reliability throughout the entire study, as the results must be as rigorous and trustworthy as possible (Bengtsson, 2016). Finally, to enhance the trustworthiness and address potential bias of the directed QCA study, three phases of preparation, organization, and reporting were also administered (Elo et al., 2014). Sample extracts from this coding process are included in Appendix A.

To understand tripartite processes and interactions, research was carried out comprehensively using WCSS22 documents and QCA, providing insight on how policy was collectively formulated and designed to address human rights issues and actor contributions. However, although documents provide rich understanding and meaning as a standalone research method (Bowen, 2009) and address a variety of questions (Hu, 2015), there are limitations to this study, being dependent on the WCSS22 narrative and researcher interpretation. Further studies could be conducted using approaches such as Qualitative Network Analysis (Ahrens, 2018), which provides additional knowledge into the meaning individual actors attach to their network ties and the network, and an insider view on the relationship. This study can, therefore, be used foundationally for further research to establish triangulation (Bowen, 2009), which might consist of extensive interviews or focus groups with tripartite policy network representatives, or external stakeholders, to gain perspectives on policy implementation.

## Findings

### Recurrent Features of the Policy Formulation and Design Process and Role of the UNGPs

**Figure 1:**
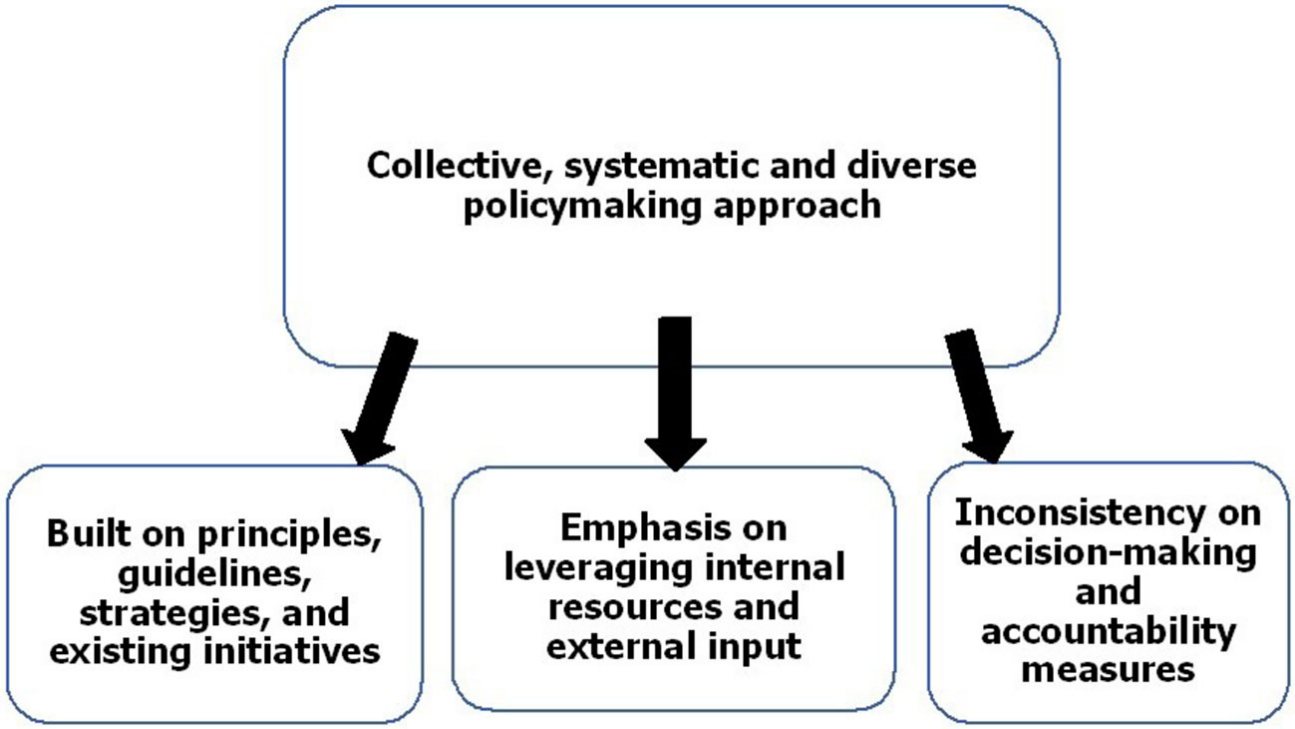
Recurrent features of policy formulation and design.

Using the analysis methods described, four recurrent features of the policy formulation and design process were identified, as well as the role of the UNGPs. These are illustrated in Figure 1.

### A Collective, Systematic, and Diverse Policymaking Approach

The tripartite or “tournament organizers” apply a collective, systematic, and diverse policy formulation approach to identify salient human rights issues and rights holder groups which could be impacted due to connected tournament activities, in accordance with the UNGPs. The term “we” is frequently applied across both strategy documents, with the tripartite policy network advocating a collective responsibility toward addressing potential impacts, providing remediation in line with the UNGPs and FIFA's Human Rights Policy, and highlighting the collective recruitment of a large workforce of migrant workers.

“*We take responsibility for addressing the impacts of the FIFA World Cup 2022*™ *from our own activities as well as those linked to our business relationships and value chains.”* **– Tournament Organizers (FIFA, 2020)**.

### Emphasis on Leveraging Internal Resources and External Input

The collaborative approach to policymaking is validated through the host country's scope of work, experience, and resources, via the participation of the SC. The strategy documents highlight a strong collaborative effort between multiple bodies is required for successful policy and strategy delivery and evolvement. The tripartite policy network addresses impact by comprehensively leveraging the best possible resources using institutions and organizations and internal (tripartite network) analysis conducted. Externally, the collective efforts also require state contribution with gaps remaining in Qatari labor regulations, despite FIFA's efforts. The stakeholder survey instructions detailed as part of the human rights assessment within the DWCSS22 also allude to factoring in the national context.

“*To be able to address the wide range of sustainability impacts of the tournament in the most effective and complete way and to leverage the best possible resources, FIFA, Q22 and the SC agreed to develop a joint sustainability strategy.”* **- Tournament Organizers (FIFA**, **2020b****)**.

### Built on Principles, Guidelines, Strategies, and Existing Initiatives

Principles such as inclusivity, integrity, transparency, responsibility, and respect for human rights guide the way the tripartite policy network delivers their joint commitments. Prioritization is also given to aspects of human rights which were already captured in the SC's strategies, FIFA handbooks or previous events, e.g., World Cups. Policy design is carefully aligned to requirements for ISO20121 amongst other national and international guidelines such as the UNGPs. Alignment with the SDGs comes through identifying and selecting relevant topics to the sustainability of the tournament and defining policy statements and objectives. Specific reference is made to collaborating to contribute as a collective working toward initiatives under each goal such as ensuring decent working and living conditions and fair recruitment for workers under Goal 8- Decent Work and Economic Growth. However, despite collective action and responsibility, there are several instances of unilateral actions by policy actors and continuance of existing initiatives such as the worker's technical cooperation program and work with contractors to reimburse fees (SC).

### Inconsistency Concerning Decision-Making and Accountability Measures

A “culture of compliance” is a phrase used consistently to provide transparency and accountability to stakeholders through decision-making and performance. However, independent experts are utilized for the human rights assessment to “gain insight of typical perspectives” instead of engaging with affected stakeholders directly. While separately, engagement is intended with those most affected to discuss progress in policy implementation. In terms of accountability, no defined outcomes are outlined, with limited detail provided regarding the “Sustainability Action Plan” for key responsibilities and KPIs, and Sustainability Management System (SMS) for monitoring process conformance, ensuring integrity and stakeholder involvement.

“*We will establish and continually improve a sustainability management system (SMS) to ensure that we fulfill our obligations…. we will develop effective, accountable and transparent institutions by maintaining an SMS.”*- **Tournament organizers (FIFA**, **2020b****)**.

### FIFA and Policy Actor Human Rights Positioning and Contribution

**Figure 2:**
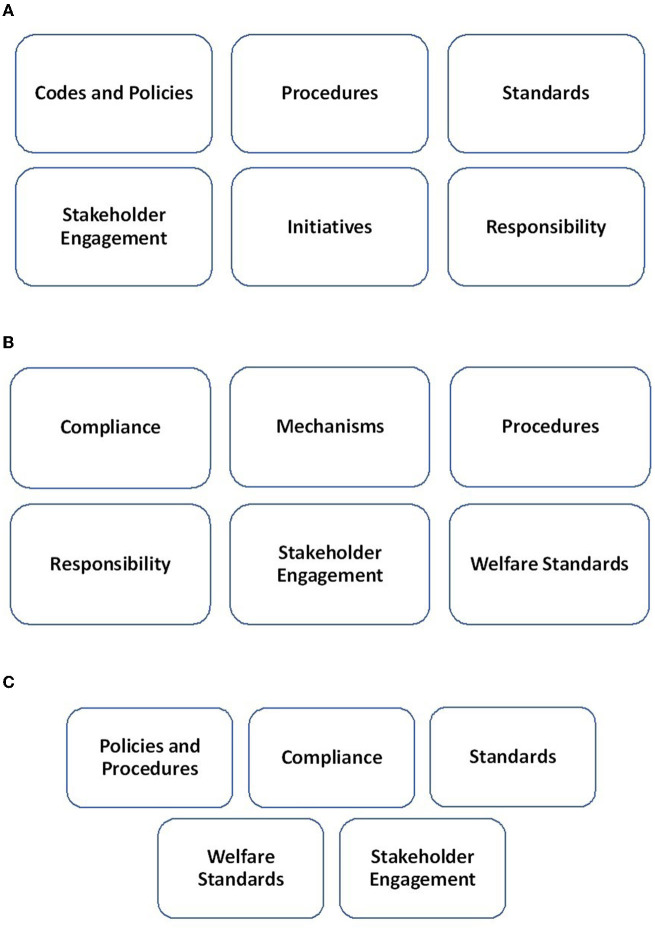
**(A)** FIFA human rights pillars. **(B)** The SC human rights pillars. **(C)** Q22 Human rights pillars.

The analysis conducted resulted in the identification of policy actor contribution and positioning in relation to human rights throughout the policymaking process. Figures 2A–C illustrate the human rights pillars for each member of the tripartite policy network of actors.

FIFA are highlighted in the strategy documents as the ultimate decision maker and contribute primarily through their existing statutes, human rights policy, and commitments to mitigate negative human rights impacts. FIFA also stated in the WCSS22 their position to champion human rights, underlining procedures for their own staff, ensuring respect by business partners, and upholding the highest international labor standards and principles with a pledge to safeguard the rights and welfare of workers. However, specified actions, and mechanisms for tournament related construction workers' living and working conditions and recruitment processes, are articulated through SC initiatives. Despite this, in accordance with the UNGPs, FIFA prioritizes the protection of human rights defenders, a group of persons whose rights may be at particular risk due to the very nature of their work, and media representatives and emphasizes the increasing inclusion of human rights-related clauses in tournament contracts.

“*Reinforcing FIFA's commitments, we pledge to safeguard the rights and welfare of workers engaged on FIFA World Cup 2022*™ *sites and to promote their rights in projects and supply chains directly linked to the FIFA World Cup.”* – **FIFA Secretary General (FIFA**, **2020b****)**.

The SC (Supreme Committee for Delivery and Legacy), the lead Qatari government entity responsible for the delivery of the tournament stadiums and infrastructure and associated services, takes a more prominent role regarding workers' rights, working and living conditions, and recruitment processes and standards. The SC attempts to be measure driven, extensive, and transparent in their strategic approach through external compliance and public progress reports. The SC also highlights their accountability and efforts to address complaints using effective grievance and remedy mechanisms, having implemented several requirements for contractors to detail recruitment and accommodation arrangements for associated workers. Furthermore, the entity, adhering to FIFA's human rights and equality statutes, advocates their Workers' Welfare Standards.

“*The SC holds itself and its partners accountable to the Workers' Welfare Standards, regularly monitoring adherence to them, and immediately addressing any cases where a party falls short.” -* **The SC (FIFA**, **2020b****)**.

Q22 is a limited liability company incorporated by FIFA and the Qatar 2022 Local Organizing Committee, responsible for the planning and delivery of operations. Although involved in collective action, Q22's input within the strategy is limited and primarily procedure driven, establishing several policies which reflect their responsibility and commitment to operate in an ethical manner, consistent with best practices locally and high standards internationally. Q22 are also members of a workers' welfare stakeholder group to holistically tackle the issues faced by workers. Finally, the Senior Sustainability Manager, part of the Q22 team, is also tasked with coordinating the related strategy development efforts.

### Exhibited Form of Network Governance in the Policy Making Process

**Figure 3:**
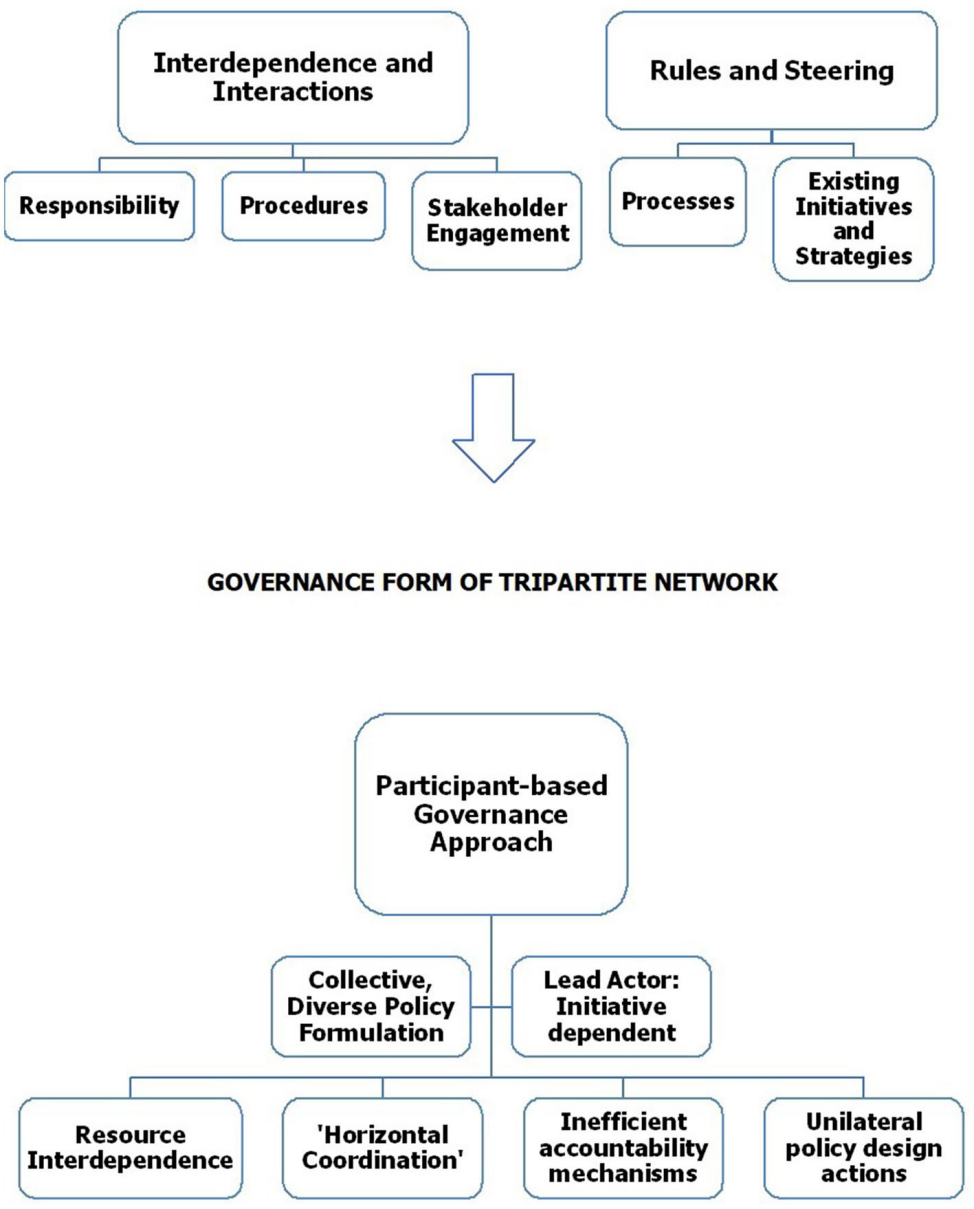
Governance form of tripartite network.

Network governance as identified in the literature (Rhodes, 2007; Fawcett and Daugbjerg, 2012; Klijn and Koppenjan, 2012), is used to ascertain its utility to explore the human rights related WCSS22 policymaking process. This in turn, assisted to identify the governance form taken. Figure 3 illustrates the connection between governance and the human rights pillars revealed from the analysis to establish the governance form of the tripartite network.

### Interdependence and Interactions

Interdependencies exist through the consensus required for actions taken and the delivery of the strategy, with each actor in the tripartite network involved in policy and strategy approval. The WCSS22 Steering Group represents an integrated approach, composed of senior executives from the tripartite network, and having overarching responsibility for reviewing performance and providing adequate resources. The WCSS22 Working Group, composed of sustainability experts from FIFA and the SC, manages the implementation of strategy and policy and provides support to project teams. However, representatives and associated tripartite network actors are not included. Interactive processes are frequent, with experts from the tripartite engaging in one-to-one meetings to build awareness of the strategy development process and discuss strategy topics, while senior executives also review and provide their input. In addition, for the human rights survey conducted as part of the strategy development process, employees of the tripartite network actors were asked to rate the level of influence of the tournament organizers to change the impacts on those most affected because of their activities. Special focus groups were carried out by actors in the tripartite network with Qatari nationals working for the SC.

“*…a meeting was held with all members of the Sustainability Steering Group to approve the full sustainability strategy, and both the FIFA Secretary General and Q22 Chairman/SC Secretary General approved and signed off on the final document.”* **- Tournament Organizers (FIFA**, **2020a****)**.

### Regulated Rules and Steering

In conjunction with carrying out assessment processes, commitments are made collectively by all actors to comply with policies and procedures in addressing potential negative human rights breaches caused by their activities. All tripartite network actors are involved in analyzing the context, identifying the initial human rights that may be breached, and coordinating the mechanisms to ascertain stakeholder feedback. Although coordination is within their remit, obscurity exists regarding the capacity of the Senior Sustainability Manager with a limited role description provided. However, the stated goals of the state and Qatar's national development strategies and goals, QNV and National Development Strategy (NDS), are claimed to be instrumental in the alignment of priorities, pillars, and objectives for the WCSS22.

“*FIFA World Cup 2022*™ *is to serve as a catalyst for the achievement of Qatar's development goals…which define the long-term outcomes for the country” -* **Chairman of the SC (FIFA**, **2020a****)**.

## Discussion

The findings from this study present distinct features of human rights' policy formulation and design, identify actor positioning and contribution concerning human rights, and illuminate the governance form in the tripartite network. The findings are discussed below with reference to existing literature, detailing their implications, and considerations for further study. Firstly, in addressing human rights through policy formulation and design, the tripartite network adopts a collective approach through systematic, integrated, and diverse mechanisms. Rather than creating discrete and distinct stages, the process for policy formulation for the WCSS22 is systematically integrated (Kim, 2006; Hermans and Cunningham, 2013; Hudson et al., 2019; Haelg et al., 2020), and coalition-based, with actors using their resources and advocating for specific policy design elements and actions based on assessment (Thatcher, 1998; Füg, 2011; Haelg et al., 2020). Collective responsibility is also adopted to identify causation or contribution to such impacts, and to provide for or cooperate in their remediation through legitimate processes, in line with the UNGPs and FIFA's Human Rights Policy (Baumann-Pauly and Nolan, 2016; Karp, 2020). From a policy network perspective, the collective approach by the tournament organizers indicates cohesive and interconnected network attributes, with actors inclined to self-identify as a cluster (Bressers and O'Toole Jr, 1998), through continued use of the term “we.” This is particularly evident for the dedicated segment related to SDG alignment, with emphasis placed on a collaborative effort to establish meaningful contribution. This collective action, as Carlsson (2000) highlights, is a way of distributing the tasks among different actors and the creation of an intelligent conformity, or coordination to guide the activities performed.

External input and internal resources are also leveraged and utilized to construct the policy commitments made. The participation of the SC adds weight to policymaking due to its scope of work, experience, and resources. However, significant emphasis is placed on leveraging internal resources and external input to develop knowledge, create objectives and policy commitments, influence, and ultimately achieve goal execution (Capano, 2018). As Klijn (2008) states, mutual dependencies emerge because actors do not themselves possess enough resources for survival or for the achievement of goals. The emergence of the tripartite network configuration appears deliberately and consciously formed to bring together the dispersed resources of network actors as suggested by Harini and Thomas (2020). Externally, joint research mechanisms (Klijn and Koppenjan, 2012), are conducted with extensive stakeholder input needed to develop a framework for the identification of salient sustainability and human rights topics, rights holder groups at risk and initiatives to address cases (Taeihagh, 2017). State contribution is also a factor with gaps remaining in Qatari labor regulations (Mares, 2011; Wettstein, 2015). However, sustainable development is complex and can involve network management practices to address multiple stakeholders, as addressed by Van Zeijl-Rozema et al. (2008) and Klijn and Koppenjan (2012).

Best practice principles and guidelines, previous and current tripartite network strategies and alignment with existing initiatives play a key role in the policymaking process. Although networks can be strongly integrated, with high levels of coordination, individual participants may also have varying interests (Carlsson, 2000) with many instances of unilateral initiatives by WCSS22 actors apparent due to their access and resources to implement (Rhodes and Marsh, 1992). The continuance of existing initiatives involving individual actors, although pertinent due to assessment feedback, might be interpreted as an issue of policy development where new elements are added to the policy mix without the removal of older ones, while existing elements are stretched to try to fit new goals and changing circumstances, as covered by Howlett and Mukherjee (2014). Nevertheless, revising existing programs or initiatives may be more sensible, achievable, and efficient, and thus may produce better policy in the long run (Peters, 2018). The state, however, plays a crucial role for policy commitments, pillars, and objectives for the WCSS2022 through alignment with Qatar's national development strategies and goals. As Rhodes (2007) and Fawcett and Daugbjerg (2012) highlight, networks are not accountable to the state, however the state can indirectly and imperfectly steer networks.

Inconsistencies exist regarding decision-making and accountability measures. Though the policy actors use best practice principles and guidelines, whilst drawing from previous and existing strategies, as was highlighted in the work of Capano (2018), there are inconsistencies concerning decision-making and accountability measures. Democratic legitimacy and greater accountability, it is argued, can be created through meaningful engagement with the wider public (Thompson and Pforr, 2005) and affected stakeholders (Amis, 2017). However, in this study, the decision was made to consult with independent experts rather than engage with affected stakeholders directly for the human rights assessment, to gain insight of typical perspectives of rights holder groups. One might argue that although the importance of experts for contributing to policy-relevant ideas can bring recognized expertise and competence in a particular realm and policy-relevant knowledge (Kisby, 2007), transparency about choices made regarding who to involve, and how the expertise is integrated into a judgement, are essential to be able to evaluate the applicability and possible biases in expert consultations (Fischer et al., 2014). Relating back to conformity, detailed by Carlsson (2000), specified targets or measures also allow policy resources to be directed toward goal attainment (Howlett, 2009). However, despite references to the Sustainability Action Plan and Sustainability Management System (SMS) for the WCSS22, which aimed to monitor conformance to processes and outcomes achieved, responsibilities and KPIs, the details of both are insubstantial. From a policy network perspective, this may have implications regarding maintaining trust and relevancy, with accountability fundamental to give account to network actors and the community (Voets et al., 2008).

FIFA's strategy to policymaking for the WCSS22, and their positioning on addressing human rights, primarily focuses on initiatives incorporating their existing codes and policies, procedures, standards, and strengthening stakeholder relationships, with emphasis placed on the importance of relationship building within a network structure in the work of Van Waarden (1992) and Hermans and Cunningham (2013). The organization states their position on the protection of human rights, however measures for tournament construction workers, with respect to living and working conditions and recruitment policies, fall dominantly under SC initiatives. This relates strongly to the argument made by Heerdt (2018) who claims any attempt to establish responsibility or accountability for major sport event-related human rights violations have either been unsuccessful or only addressed by a fraction of the actors. Thus, uncertainty prevails regarding how FIFA frames their corporate responsibility for the treatment of workers due to a lack of direct intervention in connected initiatives, as was indicated by Kirschner (2019). This, arguably, contradicts their commitments to deliver long-term positive impacts (Woods and Stokes, 2019), with FIFA's policy and principle driven approach lacking processes of enforceability and monitoring (Millward, 2017; Næss, 2018; McGillivray et al., 2019). It would appear, from a network perspective, the SC have the administrative and financial resources at their disposal (Rhodes and Marsh, 1992; Olsson and Hysing, 2012), particularly through government connection, to influence direct action for associated workers. FIFA interacts, therefore, to optimize their mutual relationship and resources through policymaking collaboration, creating a more community-based structure, a structure described in the work of Rhodes and Marsh (1992).

SC's positioning is, arguably, aimed at enhancing transparency and gaining legitimacy in their strategic approach, acting extensively to justify their authority (Provan and Kenis, 2007; Bijlmakers, 2013) on the issue of the treatment of workers connected to their activities. Substantive instruments are predominant (Howlett and Mukherjee, 2014) through their Workers' Welfare Standards and grievance mechanisms. However, the procedural instrument of network structure, as highlighted by Mukherjee et al. (2021), may not be applicable in this case, with specific measures for tournament construction workers linked directly to SC operations, rather than being network centered. Despite being the Local Organizing Committee and contributing to collective action, with the Senior Sustainability Manager on their staff, Q22's input is primarily peripheral (Rhodes and Marsh, 1992), and procedure driven, establishing numerous policies which reflect their responsibility and commitment to operate ethically (Wettstein, 2015).

Interdependencies and interactions are commonplace and deliberative throughout the policymaking process (Rhodes, 2007; Van Zeijl-Rozema et al., 2008; Fawcett and Daugbjerg, 2012; Klijn and Koppenjan, 2012), underlined by the unified, formalized, and enhanced approach to sustainability management for the tournament via the WCSS22 Steering Group and Working Groups. The interdependent nature of the tripartite network is evident through their resource driven approach (Klijn, 2008) to address the salient human rights topics, with cooperation from other institutions and organizations. Regarding regulated rules, all tripartite network actors work extensively and collectively in the processes of analyzing the context and initial topics as well as the mechanisms to formulate policy. The notion of “horizontal coordination” (Fawcett and Daugbjerg, 2012; Hermans and Cunningham, 2013) seems appropriate, with the limited number of actors for the tripartite network resulting in agreement as to the “rules of the game” indicating a relative stable network (Fawcett and Daugbjerg, 2012). However, the impact and influence of the state is evident due to clear pillar alignment with national development strategies, with the state acting as a regulator that organizes the arena in which the tripartite operates (Voets et al., 2008). As Fawcett and Daugbjerg (2012) highlight, states continue to play a central political role in setting the ground rules and context within which governance takes place. However, because of the contingent approach (Fawcett and Daugbjerg, 2012) taken for the WCSS22, and due to insufficient accountability instruments, the tripartite network may be difficult to steer or control and determine who is in charge (Keast et al., 2006). Furthermore, the complexity associated with the network and interdependence may also impede policy success by diffusing the authority and accountability mechanisms (Hale, 2011).

Finally, FIFA is noted as the lead organization concerning ownership of the tournament and the primary decision maker (Millward, 2017), with the other actors of the tripartite network adhering to FIFA statutes. However, the organization, arguably, does not have sufficient resources or control over all major network-level activities to characterize a lead governing structure (Provan and Kenis, 2007) for the tripartite. A participant-based governance form appears relevant to the study, with organizations composing the network collectively working to make both strategic and operational decisions (Provan et al., 2007). Networked power is specific to the “program” of each network (Castells, 2011, cited in Millward, 2017, p. 761), with the lead network role for the WCSS22 dependent on actor initiatives and commitments due to unilateral actions (Provan and Kenis, 2007; Howlett, 2009; Ingold et al., 2021). Based on the findings of the study, there is evidence to suggest this conceptual model of governance can be used for other studies of policy networks in mega sport events, via methods that capture the network partners dialogue throughout the policy formation process.

## Conclusion

This study aimed to understand the role played by FIFA, and other actors, as they looked to address human rights through policy formulation and design for the WCSS22, using UNGP alignment, and a policy network theoretical framework. In addition, this study also intended to identify the governance form established. The findings highlight a cohesive and coalition constructed policy formulation approach, anchored through leveraging tripartite network resources and extensive stakeholder input. Policy design is produced using the building foundation of best practice principles, guidelines, strategies, and existing initiatives; however, unilateral actor actions are prominent with inconsistencies concerning decision-making and accountability measures. In terms of policy actor position and contribution, FIFA focus on their existing statutes, human rights policy, and commitments to mitigate negative human rights impacts. However, their efforts appear to lack enforceability with specified measures for tournament construction workers' conditions, articulated through the SC's initiatives. The SC's positioning is, arguably, based on enhancing transparency and gaining legitimacy, and projecting their accountability, using their resources to take a more prominent role concerning worker initiatives. Despite involvement in collective action, and a workers' welfare stakeholder group, Q22's input is predominantly peripheral. Interdependencies and interactions are commonplace, through both the WCSS22 Steering and Working Groups, with state contribution influential, through alignment with their national development strategies. Due to insufficient accountability mechanisms, the tripartite network demonstrates difficulties in determining those steering and responsible. This bodes ill for the capacity of the governance form to deliver the FIFA World Cup Qatar 2022 at the level of the UNGPs standard. With varied resources at their disposal, a participant-based governance form was identified with the lead role dependent on actor initiatives and commitments. In summary, the findings from the study highlight the significance and relevance of the tripartite network in relation to human rights and further enhance our understanding of network policymaking processes and actor interrelations and interactions.

## Data Availability Statement

The raw data supporting the conclusions of this article will be made available by the authors, without undue reservation.

## Author Contributions

Both authors listed have made a substantial, direct, and intellectual contribution to the work and approved it for publication.

## Funding

This work was supported by Brandon Shad RE (Fee Support Program).

## Conflict of Interest

The authors declare that the research was conducted in the absence of any commercial or financial relationships that could be construed as a potential conflict of interest.

## Publisher's Note

All claims expressed in this article are solely those of the authors and do not necessarily represent those of their affiliated organizations, or those of the publisher, the editors and the reviewers. Any product that may be evaluated in this article, or claim that may be made by its manufacturer, is not guaranteed or endorsed by the publisher.
