# Supplementary material for: The FIFA World Cup Qatar 2022 Sustainability Strategy: Human Rights Governance in the Tripartite Network
Source: Front Sports Act Living. 2022 May 23;4:809984. doi: 10.3389/fspor.2022.809984 (PMC9168318; doi:10.3389/fspor.2022.809984)
Supplement: Supplementary file 1 [file Table_1.docx]

**APPENDIX A**

**Samples of Coding for Recurrent Features of Policy Formulation and Design:**

| **Extract** | **Open Code** | **Theme Categorization** | **Subcategory** | **Main Categorization/Feature** |
| --- | --- | --- | --- | --- |
| *“Through this strategy, we seek to align our efforts*  *to advance sustainable development with the UN’s*  *Sustainable Development Goals (SDGs) that aim to*  *protect the planet and ensure that all people enjoy*  *peace and prosperity by 2030”* | Within the strategy, alignment of efforts for sustainability using the SDGs | SDG alignment | Aligning goals | **Built on principles, guidelines, strategies, and existing initiatives** |
| *“The progress achieved by the government of*  *Qatar will facilitate our efforts to ensure the welfare of*  *all workers associated with the tournament in Qatar, in*  *particular those engaged by entities over which we lack*  *direct control.”* | Progress by government will facilitate efforts to ensure welfare of workers where there is a lack of direct control | Government efforts facilitate tripartite network | External facilitation | **Emphasis on leveraging internal resources and external input** |
| *“In addition, more than 20 external stakeholder representatives and more than 50 experts from FIFA, Q22 and the SC were engaged in one-to-one meetings to build awareness of the strategy development process and to discuss particular strategy topics.”* | External stakeholder representatives and experts from tripartite engaged with to build awareness and discussion | External stakeholders and internal experts utilized | Stakeholder involvement | **A collective, systematic, and diverse policymaking approach** |
| *“Sustainability governance:*  *Establishing clear roles, responsibilities, and governance structures for sustainability, including at*  *board level and for the Sustainability Steering Group, and ensuring that these are well-documented,*  *implemented and reviewed.”* | Sustainability governance -clear roles, structures for sustainability, documented, and implemented | Clear role clarification and implementation | Accountability | **Inconsistency concerning decision-making and accountability measures** |

**Samples of Coding for Actor Contribution**

| **Extract** | **Open Code** | **Theme Categorization** | **Subcategory** | **Key Human Rights Positioning Pillars for each actor** |
| --- | --- | --- | --- | --- |
| *“FIFA has thereby urged the Qatari government to take decisive steps to enhance the*  *protection of migrant workers in accordance with relevant international standards and recognizes the significant commitments and progress made in recent years.”* | FIFA urged Qatari government to enhance protection of workers | Government urged to enhance protection | Government involvement | **Stakeholder Engagement** |
| *“The standards clearly set out*  *the SC’s requirements regarding the recruitment,*  *employment, living and working conditions for*  *everyone engaged on an SC site, and will evolve over*  *time as working practices change.”* | Standards clearly set out SC requirements regarding recruitment, employment, and conditions | SC requirements regarding recruitment, employment | SC requirements | **Responsibility** |
| *Q22 and the SC have each established a number*  *of policies which reflect their responsibility and*  *commitment to operate in an ethical manner,*  *consistent with both best practices in the*  *local market and the high standards set by the*  *international community.* | Q22 and SC established policies to reflect responsibility and commitment to operate in ethical manner, with high standards set | Q22 and SC established policies to reflect responsibility to ethics | Q22 and SC policies | **Codes & Policies** |

**Sample of coding to ascertain Governance Form**

| **Key Positioning Pillars** | **Conceptual Model Component** | **Definition/Explanation** | **Tripartite Network Governance Form and Summary of Findings** |
| --- | --- | --- | --- |
| Responsibility, Procedures and Codes and Policies | Interdependence | “Policy and service delivery are formed and  implemented in a network of interdependent actors. Most network researchers  agree that interdependency is the core factor that initiates and sustains networks.” (Klijn and Koppenjan, 2012; p.591) | **Participant-based Governance Approach:**   - Collective, Diverse Policy Formulation - Resource Interdependence - 'Horizontal Coordination’ - Lead Actor: Initiative dependent - Unilateral policy design actions - Inefficient accountability mechanisms |
| Stakeholder Engagement | Interactions | “The set of interactions define  who will become core members of a network, who will be positioned at its periphery  with occasional and usually limited influence.” (Fawcett and Daugbjerg, 2012; p.198) |  |
| Processes | Regulated Rules | “Game-like interactions, rooted in trust and regulated by rules of the game  negotiated and agreed by network participants.” (Rhodes, 2007; p.4) |  |
| Existing Initiatives and Strategies | Steering | “Networks are not  accountable to the state; they are self-organizing. Although the state does  not occupy a privileged, sovereign position, it can indirectly and imperfectly  steer networks (Rhodes, 2007; p.4) |  |
